# Genetic diversity and molecular characterization of avian paramyxoviruses from wild birds in South Korea between 2020 and 2024

**DOI:** 10.3389/fmicb.2026.1732075

**Published:** 2026-02-17

**Authors:** Eun-Jee Na, Su-Beom Chae, Young-Sik Kim, Jun-Soo Park, Serin Sim, Seung-Chai Kim, Hwan-Ju Kim, Chang-Gi Jeong, Jae-Ku Oem

**Affiliations:** 1 Laboratory of Veterinary Infectious Disease, College of Veterinary Medicine, Jeonbuk National University, Iksan, Republic of Korea; 2 College of Veterinary Medicine, Jeonbuk National University, Iksan, Republic of Korea; 3 Biosafety Research Institute and College of Veterinary Medicine, Jeonbuk National University, Iksan, Republic of Korea

**Keywords:** wild bird, avian paramyxovirus, APMV-1, APMV-4, APMV-6, APMV-13, SISPA

## Abstract

Avian paramyxoviruses (APMVs) are economically important pathogens in the poultry industry, and wild waterfowl are considered reservoirs contributing to the spread of APMVs. In the present study, we investigated the prevalence and molecular characterization of APMVs in wild birds in South Korea between September 2020 and February 2024. A total of 27 APMVs were obtained, with a mean isolation rate of 0.35%. The APMV serotypes were identified as APMV-1 (*n* = 12), APMV-4 (*n* = 13), APMV-6 (*n* = 1), and APMV-13 (*n* = 1). Viral RNA genomes were amplified, and nearly complete genomic sequences were recovered using the single-primer amplification (SISPA) approach. All APMV isolates possessed a monobasic cleavage site in the fusion (F) protein, suggesting that they are low-pathogenic strains. Phylogenetic analysis based on complete F protein sequences revealed that APMV-1 isolates classified into class I sub-genotype 1.2 and class II sub-genotype I.2. Notably, APMV-1 isolates within class I sub-genotype 1.2 exhibited several amino acid substitutions compared to Newcastle disease virus (NDV) vaccine strains. To our knowledge, this study provides the first molecular evidence for the presence of a genotype II APMV-4 strain in wild birds from South Korea, suggesting the considerable genetic diversity of APMVs currently circulating among wild bird populations. Taken together, the nearly complete genomic sequences and genetic characterization of diverse APMV subtypes obtained in this study provide a valuable foundation for future research on their molecular evolution, antigenic variation, and epidemiological behavior.

## 1 Introduction

Avian paramyxoviruses (APMVs) belong to the subfamily *Avulavirinae*, family *Paramyxoviridae*, and currently define 22 different avulavirus species (APMV-1 to APMV-22) that are further divided into three genera. *Metaavulavirus* (APMV-2, -5 to -8, -10, -11, -14, -15, -20, and -22), *Orthoavulavirus* (APMV-1, -9, -12, -13, -16 to -19, and -21), and *Paraavulavirus* (APMV-3 and -4) according to the International Committee on Taxonomy of Viruses (ICTV) (Young et al., 2022). APMVs are negative-sense, single-stranded RNA viruses (Lee et al., 2017). Most APMVs encode at least six proteins, namely nucleoprotein (N), phosphoprotein (P), matrix (M) protein, fusion (F) protein, hemagglutinin-neuraminidase (HN) protein, and large polymerase (L), which are arranged in the order of 3’ leader, N, P, M, F, HN, L and 5’ trailer (Samal, 2011). Among these, the F protein is involved with the entry of the virus, and the HN protein has receptor-binding and neuraminidase activity and contributes to viral replication (Kim et al., 2011; Morrison, 2003). Partial or complete nucleotide sequences of the F gene have been commonly employed in phylogenetic analyses for genotype classification (Diel et al., 2012).

Specific APMV serotypes are important in the poultry industry due to their ability to cause clinical disease in domestic birds (Saif, 2009). APMV-1, known as Newcastle disease virus (NDV), is globally distributed, and its highly pathogenic forms cause substantial economic losses to the poultry industry (Alexander et al., 2012). APMV-1 is classified based on clinical severity and molecular characterization of the amino acid sequences at the F protein cleavage site. Velogenic strains, characterized by high virulence, possess a multi-basic cleavage site motif, whereas lentogenic strains exhibit low virulence and contain a monobasic motif (Seal et al., 2005; Klink et al., 2023). While the remaining APMV serotypes are generally regarded as low or asymptomatic virulence in poultry, APMV-2, APMV-3, APMV-4, APMV-6, and APMV-7 have been reported to cause mild clinical signs in domestic birds (Nayak et al., 2012; Yin et al., 2017; Alexander, 2000).

Wild birds are known as natural reservoirs of both APMVs and influenza A viruses (Thomas et al., 2008). During seasonal migration, wild birds play a pivotal role in the intercontinental dissemination of viruses across geographic regions (Treshchalina et al., 2022). To date, nine major migratory bird flyways have been recognized worldwide, serving as critical routes for seasonal wild birds’ movements (Pearce-Higgins et al., 2017). South Korea is located in the East Asian-Australasian Flyway (EAAF), acting as a key migratory corridor and breeding site for numerous migratory bird species (Son et al., 2024). In particular, the western regions of South Korea serve as major wintering grounds for migratory birds, with increased wild bird abundance during autumn migration and winter (Lee et al., 2020). These western regions are characterized by high densities of both wild birds and poultry, creating favorable conditions for the circulation of avian influenza (AI) viruses (Song et al., 2017). In response, active AI virus surveillance programs targeting migratory birds have been conducted in South Korea since 2012, contributing to a better understanding of APMV distribution and diversity among wild birds in South Korea (Jeong et al., 2018). Several APMV serotypes, including APMV-1, APMV-4, APMV-6, APMV-13, APMV-14, APMV-15, and APMV-21, have been detected in wild waterfowl migrating through South Korea (Lee et al., 2024; Choi et al., 2013; Lee et al., 2017; Choi et al., 2018; Kuhn et al., 2020; Youk et al., 2025). Notably, lentogenic APMV-1 strains isolated from domestic ducks in South Korea exhibited high genetic similarity to virulent NDV strains previously identified in wild waterfowl in China, implying possible interregional transmission (Gaikwad et al., 2016). Furthermore, APMV-4 has been isolated not only from wild birds but also from domestic ducks, supporting the possibility of interspecies transmission between wild and domestic avian populations (Tseren-Ochir et al., 2017). These findings underscore the importance of continued surveillance of APMVs in wild birds to understand their role in APMV epidemiology better and to inform effective prevention strategies.

Recent advances in high-throughput sequencing technologies have significantly facilitated investigations into the genetic diversity and evolutionary dynamics of APMVs (Dimitrov et al., 2019). Accordingly, this study employed a next-generation sequencing (NGS) approach using single-primer amplification (SISPA) to obtain complete genome sequences and assess the genetic characteristics of APMVs from wild bird fecal samples collected in South Korea between 2020 and 2024.

## 2 Materials and methods

### 2.1 Sample collection and virus isolation

Between September 2020 and February 2024, a total of 7,677 fecal samples were collected primarily during the winter season from wild bird habitats in four western provinces of South Korea (Gyeonggi, Chungbuk, Chungnam, and Jeonbuk). The collected samples were immediately transported to the laboratory and stored at 4 °C until further processing.

The samples were suspended in phosphate-buffered saline (PBS, pH 7.4) containing 1X Gibco^®^ antibiotic–antimycotic solution (Gibco, United States) and centrifuged at 2,800 × g for 10 min. Supernatants were filtered with a 0.45 μm syringe filter (Sartorius, Germany). The filtrate was inoculated into the allantoic cavities of 9–11-day-old specific-pathogen-free (SPF) embryonated chicken eggs (Seong-Min Inc., Korea). After 5 days at 37°C, the allantoic fluids were harvested and tested by a hemagglutination assay (HA). Viral RNA was extracted from HA-positive allantoic fluids using QIAamp Viral RNA Mini Kit (Qiagen, Germany). The extracted viral RNA was tested for AIV using the LiliF AIV M real-time reverse transcription polymerase chain reaction (RT-PCR) Kit (iNtRON Biotechnology, Korea) and for APMV by reverse-transcription polymerase chain reaction (RT-PCR) as previously described (Tong et al., 2008; Na et al., 2022).

### 2.2 Host identification

The genomic DNA was extracted from APMV-positive fecal samples using the QIAamp DNA mini Kit (Qiagen, Germany). Host species were identified from fecal samples based on mitochondrial cytochrome C oxidase subunit I (COI) gene sequence, following previously established methods (Lee et al., 2010).

### 2.3 Nucleotide sequencing

Viral RNA that tested positive for APMV by RT-PCR was cleaned up using RNAClean XP beads (Beckman Coulter, United States) and eluted in DEPC-treated water. The quantity and quality of the RNA were assessed using Qubit™ RNA HS Assay Kit (Thermo Fisher Scientific, United States) on a Qubit 4 Fluorometer (Thermo Fisher Scientific, United States). First-strand complementary DNA (cDNA) was synthesized with the SuperScript IV First-Strand Synthesis System (Invitrogen, United States) with K-6N SISPA primer (K-6N: 5’-GACCATCTAGCGACCTCCACNNNNNN-3’), following previously described protocols (Rosseel et al., 2013). For second-strand cDNA synthesis, the first-strand cDNA was incubated at 22 °C for 30 min with Klenow Fragment I (NEB, United States), NEBuffer™ 2 (NEB, United States), and the same K-6N SISPA primer. The synthesized double-stranded DNA was purified using AMPure XP beads (Beckman Coulter, United States) and used as a template for random PCR amplification with the Platinum™ SuperFi II PCR 2 × Master Mix (Thermo Fisher Scientific, United States) and primer K (K: 5’-GACCATCTAGCGACCTCCAC-3’) as described previously (Kim et al., 2022). PCR products were purified using the HiGene™ Gel and PCR Purification System (BIOFACT, Korea) and quantified using the Qubit dsDNA HS Assay (Thermo Fisher Scientific, United States).

The PCR amplifications were sent to BIONICS (South Korea), and the raw data were obtained using BIONICS index-tagged (BIT) sequencing methods (BIONICS, Korea) based on 100,000 reads. Raw data were processed using Trimmomatic (v0.39) for quality trimming and assembled de novo using SPAdes assembler (v4.0.0). The genome sequences of all obtained APMV isolates have been submitted to GenBank.

### 2.4 Genetic and phylogenetic analysis

Reference sequences used for the phylogenetic analyses were retrieved from the GenBank database. All sequences were aligned using the Clustal Omega algorithm (Sievers et al., 2011). The amino acid substitutions in APMV-1 were analyzed by comparing the V4 (accession no. JX524203) and LaSota (accession no. ON713864) strains with NDV vaccine strains. For APMV-4, amino acid substitutions were assessed by comparison with the reference strain JX-G13 (accession no. MN336346).

Phylogenetic trees were constructed with the maximum likelihood method using IQ-TREE software version 1.6.12 with 1,000 bootstrap replications (Trifinopoulos et al., 2016). The optimal substitution model for each alignment was determined using ModelFinder, integrated into IQ-TREE version 1.6.12. Phylogenetic trees were visualized using the Interactive Tree of Life (iTOL) (Letunic and Bork, 2021).

### 2.5 Mean death time assay

The virulence of seven representative APMV isolates was assessed using the mean death time (MDT) assay in 9-day-old SPF embryonated chicken eggs. Representative strains were selected from different genotypes of each APMV serotype based on phylogenetic analysis (Table 4). The MDT was performed according to established procedures (Swayne, 1998; Kim et al., 2012). Tenfold serial dilutions of infected allantoic fluid were prepared, and 0.1 mL of each dilution was inoculated into embryonated chicken eggs. Embryo mortality was monitored at 8 h intervals for 7 days to calculate MDT values. APMV isolate virulence was classified based on MDT criteria as velogenic (<60 h), mesogenic (60–90 h), and lentogenic (more than 90 h) (Grimes, 2002).

### 2.6 Hemagglutination inhibition assay

Antigenic cross-reactivity between 12 APMV-1 isolates and the NDV vaccine strain was evaluated using a hemagglutination inhibition (HI) assay with reference anti-NDV LaSota serum (Daesung Microbiological Labs Co., Korea). HI assays were conducted according to standard procedures as previously reported (Stear, 2005). Serum samples were treated with receptor-destroying enzyme (RDE) (Seiken, Japan) at 1:5 serum-RDE ratio and incubated in a 37°C water bath overnight, followed by enzyme inactivation at 56°C for 30 min. PBS was subsequently added to adjust the final serum dilution to 1:10. Serial two-fold dilutions of the treated sera were prepared and incubated with APMV-1 antigens standardized to four hemagglutinating units (HAU). After incubation at room temperature for 30 min, 1% chicken red blood cells were added, and the results were read after an additional 40 min. The HI titers were defined as the reciprocal of the highest serum dilution showing complete inhibition of hemagglutination.

## 3 Results

### 3.1 APMV detection in wild birds in South Korea

Between September 2020 and February 2024, 27 APMV isolates (0.35%) were obtained from 7,677 fresh wild bird fecal samples in South Korea. Five out of 2,499 samples (0.20%) were isolated as APMV between September 2020 and March 2021. In the following surveillance periods, 3 of 1,343 samples (0.22%) were isolated between November 2021 and January 2022, 5 of 1,866 samples (0.26%) between October 2022 and February 2023, and 14 of 1,969 samples (0.71%) between October 2023 and March 2024. Of the 27 APMV isolates, host species identification was unsuccessful for 10 strains. The remaining 17 APMV strains all belonged to the order *Anseriformes*. Among these, 14 were isolated from *Anas platyrhynchos* (Mallard), one each from *Spatula clypeata* (Northern Shoveler), *Mareca strepera* (Gadwall), and *Mareca penelope* (Eurasian wigeon) (Table 1).

**TABLE 1:** APMVs isolated in South Korea from 2020 to 2024.

Isolate	Abbreviation	Year	Location	Species	Serotype	Fusion gene cleavage site	Accession number
Wild bird/South Korea/20JBN-F12-31-35/2020	20JBN-F12-31-35	2020	Chungnam	Wild bird	APMV-1	ERQER ↓ L	PX399393
Wild bird/South Korea/20JBN-F12-41-45/2020	20JBN-F12-41-45	2020	Chungnam	Wild bird	APMV-1	ERQER ↓ L	PX399394
Wild bird/South Korea/20JBN-F12-46-50/2020	20JBN-F12-46-50	2020	Chungnam	Wild bird	APMV-1	ERQER ↓ L	PX409997
Wild bird/South Korea/20JBN-F13-21-25/2020	20JBN-F13-21-25	2020	Jeonbuk	Wild bird	APMV-1	GKQGR ↓ L	PX409998
Wild bird/South Korea/21JBN-F27-27/2021	21JBN-F27-27	2021	Jeonbuk	Wild bird	APMV-1	GKQGR ↓ L	PX409999
Wild bird/South Korea/21JBN-F1-50/2021	21JBN-F1-50	2021	Jeonbuk	Wild bird	APMV-1	GKQGR ↓ L	PX410000
*Anas platyrhynchos*/South Korea/22JBN-F12-14/2022	22JBN-F12-14	2022	Jeonbuk	*Anas platyrhynchos*	APMV-1	GKQGR ↓ L	PX410001
*Anas platyrhynchos*/South Korea/22JBN-F12-104/2022	22JBN-F12-104	2022	Jeonbuk	*Anas platyrhynchos*	APMV-4	DIQPR ↓F	PX399395
*Anas platyrhynchos*/South Korea/22JBN-F2-10/2022	22JBN-F2-10	2022	Jeonbuk	*Anas platyrhynchos*	APMV-1	ERQER ↓ L	PX410002
*Anas platyrhynchos*/South Korea/22JBN-F4-34/2022	22JBN-F4-34	2022	Jeonbuk	*Anas platyrhynchos*	APMV-6	APEPR ↓ L	PX410003
*Anas platyrhynchos*/South Korea/22JBN-F5-51/2022	22JBN-F5-51	2022	Jeonbuk	*Anas platyrhynchos*	APMV-4	DIQPR ↓F	PX399396
*Anas platyrhynchos*/South Korea/22JBN-F14-46-50/2022	22JBN-F14-46-50	2022	Jeonbuk	*Anas platyrhynchos*	APMV-13	VRENR↓L	PX410004
*Anas platyrhynchos*/South Korea/23JBN-F22-66-70/2023	23JBN-F22-66-70	2023	Jeonbuk	*Anas platyrhynchos*	APMV-1	ERQER ↓ L	PX410005
*Anas platyrhynchos x Anas superciliosa*/South Korea/23JBN-F2-1/2023	23JBN-F2-1	2023	Chungnam	*Anas platyrhynchos x Anas superciliosa*	APMV-1	ERQER ↓ L	PX410006
*Anas platyrhynchos*/South Korea/23JBN-F2-93/2023	23JBN-F2-93	2023	Chungnam	*Anas platyrhynchos*	APMV-4	DIQPR ↓F	PX399397
*Anas platyrhynchos*/South Korea/23JBN-F3-38/2023	23JBN-F3-38	2023	Jeonbuk	*Anas platyrhynchos*	APMV-4	DIQPR ↓F	PX399398
*Anas platyrhynchos*/South Korea/23JBN-F7-11/2023	23JBN-F7-11	2023	Jeonbuk	*Anas platyrhynchos*	APMV-1	GKQGR ↓ L	PX410006
*Anas platyrhynchos*/South Korea/23JBN-F7-68/2023	23JBN-F7-68	2023	Jeonbuk	*Anas platyrhynchos*	APMV-1	ERQER ↓ L	PX410008
*Anas platyrhynchos*/South Korea/23JBN-F9-76-80/2023	23JBN-F9-76-80	2023	Jeonbuk	*Anas platyrhynchos*	APMV-4	DIQPR ↓F	PX396318
Wild bird/South Korea/23JBN-F10-46-50/2023	23JBN-F10-46-50	2023	Jeonbuk	Wild bird	APMV-4	DIQPR ↓F	PX396319
*Anas platyrhynchos*/South Korea/24JBN-F15-83/2024	24JBN-F15-83	2024	Jeonbuk	*Anas platyrhynchos*	APMV-4	DIQPR ↓F	PX396320
*Spatula clypeata*/South Korea/24JBN-F17-26/2024	24JBN-F17-26	2024	Jeonbuk	*Spatula clypeata*	APMV-4	DIQPR ↓F	PX396321
*Mareca strepera*/South Korea/24JBN-F17-28/2024	24JBN-F17-28	2024	Jeonbuk	*Mareca strepera*	APMV-4	DIQPR ↓F	PX396322
Wild bird/South Korea/24JBN-F17-33/2024	24JBN-F17-33	2024	Jeonbuk	Wild bird	APMV-4	DIQPR ↓F	PX396323
Wild bird/South Korea/24JBN-F17-34/2024	24JBN-F17-34	2024	Jeonbuk	Wild bird	APMV-4	DIQPR ↓F	PX396324
*Mareca penelope*/South Korea/24JBN-F17-104/2024	24JBN-F17-104	2024	Jeonbuk	*Mareca penelope*	APMV-4	DIQPR ↓F	PX396325
Wild bird/South Korea/24JBN-F17-56-60/2024	24JBN-F17-56-60	2024	Jeonbuk	Wild bird	APMV-4	DIQPR ↓F	PX396326

### 3.2 Genetic and phylogenetic analyses of APMV isolates

Complete coding sequences (CDSs) for the six major genes (N, P, M, F, HN, and L) were successfully obtained for 25 out of the 27 APMV isolates (Supplementary Table 1). However, non-coding regions of each gene, particularly the 3’ and 5’ untranslated regions (UTRs), were partially recovered in most sequences (Supplementary Table 1). The APMV sequences obtained in this study, comprising CDSs of the six major genes with partially recovered untranslated regions, have been deposited in the GenBank database with accession numbers (Table 1).

**FIGURE 1:**
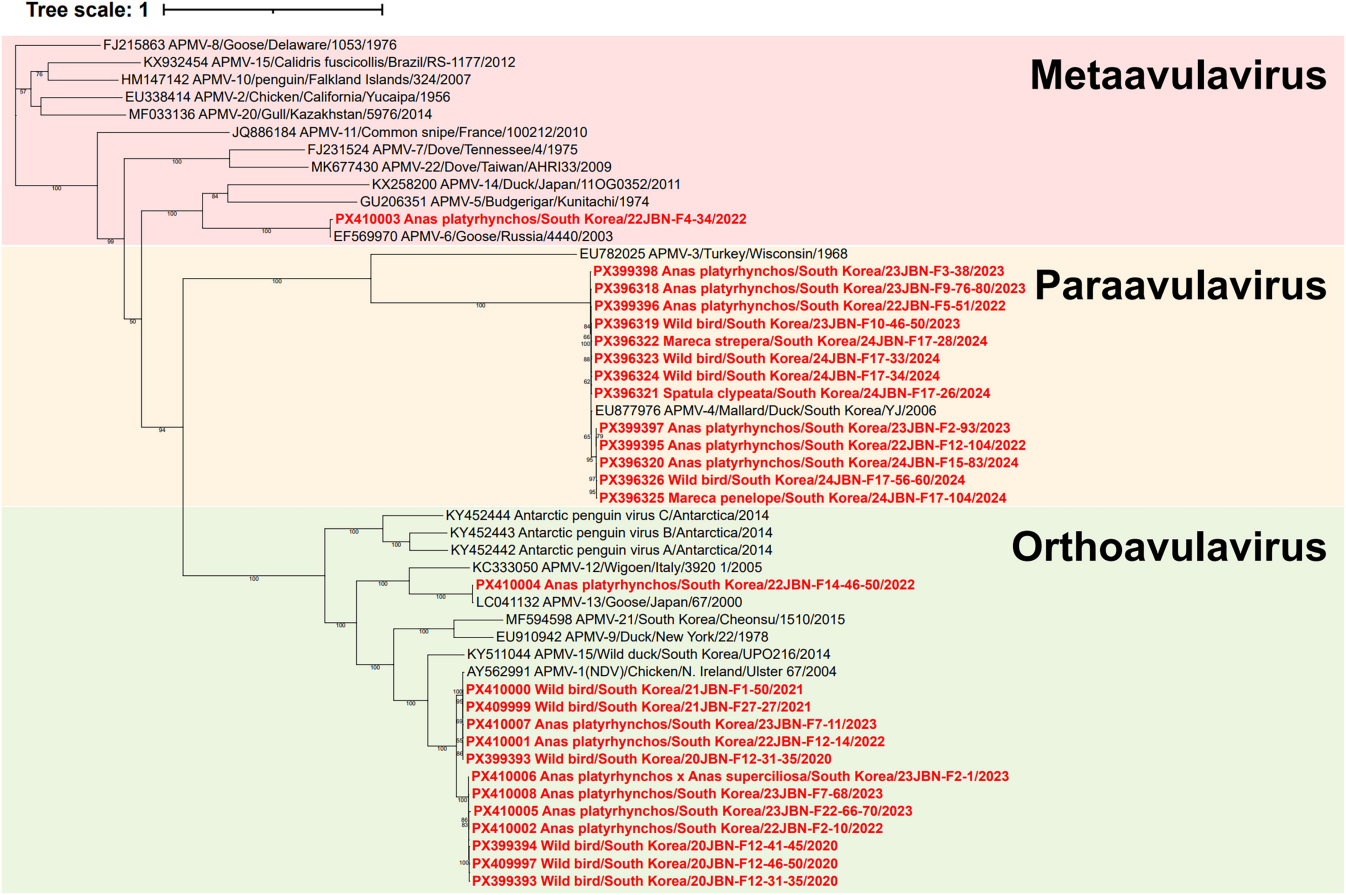
Phylogenetic analysis of complete polymerase (L) amino acid sequences of 22 reference APMVs and 27 APMVs isolated in this study. The phylogenetic tree was constructed using maximum likelihood with the LG+F+I+G4 model and supported by 1,000 bootstrap replicates. APMV strain sequences in the present study are highlighted in bold red. The reference strains include representatives from the genera *Metaavulavirus* (APMV-2, −5 to −8, −10, −11, −14, −15, −20, and −22), *Orthoavulavirus* (APMV-1, −9, −12, −13, −16 to −19, and −21), and *Paraavulavirus* (APMV-3 and −4).

Based on the current ICTV phylogenetic methodology for the *Paramyxoviridae* family, phylogenetic analysis of the complete L amino acid sequences classified the isolates into four serotypes: 12 isolates were identified as APMV-1, 13 as APMV-4, one as APMV-6, and one as APMV-13 (Figure 1 and Table 1; Rima et al., 2019). However, due to the limited availability of complete genome sequences for APMV serotypes, phylogenetic analyses of each serotype were additionally conducted using the complete nucleotide sequences of the F gene, which is commonly used for genotype classification in paramyxoviruses (Liu et al., 2022).

### 3.3 Genetic and phylogenetic characterization of APMV-1

**FIGURE 2:**
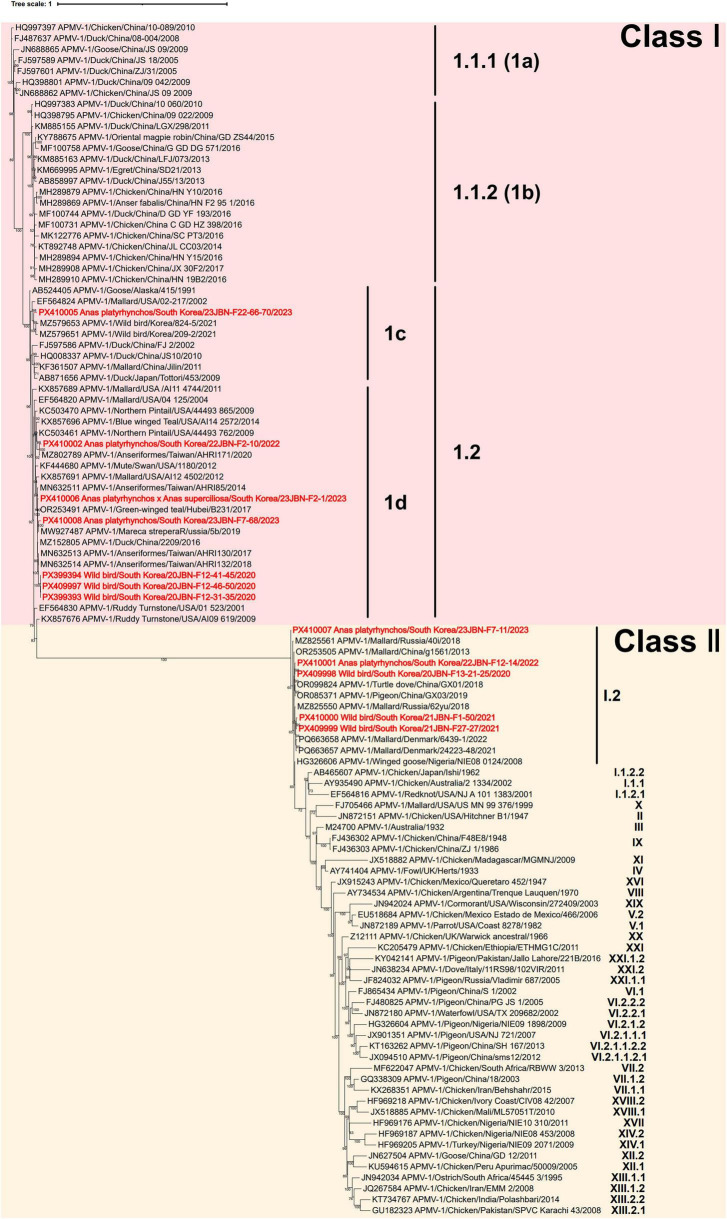
Phylogenetic analysis of the full-length nucleotide sequence of the fusion (F) gene of APMV-1 isolates. The phylogenetic tree was constructed using maximum likelihood with the GTR+F+R3 model and supported by 1,000 bootstrap replicates. APMV-1 strain sequences in the present study are highlighted in bold red. Sub-genotype classification follows the criteria established by Dimitrov et al., whereas the sub-clustering into 1a, 1b, 1c, and 1d is based on the classification proposed by Diel et al.

Seven APMV-1 isolates were grouped within class I sub-genotype 1.2, and five were classified as class II sub-genotype I.2, based on the genotyping criteria proposed by Dimitrov et al. (2019) (Figure 2). Among the seven class I sub-genotype 1.2 isolates, six were classified as sub-genotype 1d, while one isolate (23JBN-F22-66-70) was grouped as 1c, according to the classification system previously described by Diel et al. (2012). All isolates within each sub-genotype shared over 95% nucleotide identity based on the complete F gene sequence, consistent with the defined classification criteria established by Dimitrov et al. (2019). 23JBN-F22-66-70 showed the highest identity of 97.8% with Wild bird/Korea/209-2/2021. 22JBN-F-2-10 shared 98.6% identity with Northern Pintail/USA/44493 762/2009. 23JBN-F2-1 and 23JBN-F7-68 were most closely related to Green-winged Teal/Hubei/B231/2017, with 98.8% identity. Three isolates, 20JBN-F12-41-45, 20JBN-F12-46-50, and 20JBN-F21-31-35, clustered together and were closely related to Anseriformes/Taiwan/AHRI1322018, exhibiting 99.2% identity.

23JBN-F7-11 showed 99.2% identity with Mallard/Russia/40i/2018. 20JBN-F13-21-25 and 22JBN-F12-14 exhibited the highest identity (99.6%) with Pigeon/China/GX03/2019. 21JBN-F1-50 and 21JBN-F27-27 showed 99.1% and 99.3% identity with Mallard/Denmark/24223-48/2021, respectively.

The gene-start (GS) and gene-end (GE) signal sequences identified from the six major genes of the APMV-1 strain isolated in this study were 3’-UGCCCAUC(C)UU-5’ and 3’-AAUCU_6_-5’, respectively. These sequences are consistent with those reported in previous studies on APMV-1 (Samuel et al., 2010). The F protein cleavage site motif was ERQERL in class I sub-genotype 1.2 isolates and GKQGRL in class II sub-genotype I.2 isolates, both indicative of avirulent APMV strains.

Amino acid sequences of the F and HN proteins were compared with two reference vaccine strains, V4, which is an apathogenic genotype I, and LaSota, a lentogenic Genotype II, in order to identify amino acid substitutions (Table 2; Zhang et al., 2021; Elbestawy et al., 2023). In the F protein, one substitution (D170S) was observed in the heptad repeat (HR) region, two substitutions (I509T and S511A) were found in the transmembrane domain, and two substitutions (D170S and A75Q) were located within neutralizing epitopes in isolates of class I sub-genotype 1.2. In contrast, amino acids of class II sub-genotype I.2 isolates were conserved in these regions. Analysis of the conserved cysteine residues revealed that residue 27 was replaced by glycine in class I isolates and by arginine in class II isolates.

**TABLE 2:** Amino acid substitutions in the F protein of APMV-1 isolates.

Strain	Genotype	Heptad repeat (HR) region	Transmembrane domain (aa 500–522)		Cysteine residues	Neutralizing epitopes	
		D^170^	I^509^	S^511^	C^27^	D^170^	A^75^
V4	Class I	N	–	–	–	–	–
LaSota	Class II	–	V	–	–	N	–
20JBN-F12-31-35	Class I 1.2	S	T	A	G	S	Q
20JBN-F12-41-45	Class I 1.2	S	T	A	G	S	Q
20JBN-F12-46-50	Class I 1.2	S	T	A	G	S	Q
20JBN-F13-21-25	Class II I.2	–	V	–	R	–	–
21JBN-F27-27	Class II I.2	–	V	–	R	–	–
21JBN-F1-50	Class II I.2	–	V	–	R	–	–
22JBN-F12-14	Class II I.2	–	V	–	R	–	–
22JBN-F2-10	Class I 1.2	S	T	A	G	S	Q
23JBN-F22-66-70	Class I 1.2	S	T	A	G	S	Q
23JBN-F2-1	Class I 1.2	S	T	A	G	S	Q
23JBN-F7-11	Class II I.2	–	V	-	R	–	–
23JBN-F7-68	Class I 1.2	S	T	A	G	S	Q

Heptad repeat (HR) region is defined as HR-A (aa 143–185), HR-B (aa 268–289), and HR-C (aa 467–502). The Transmembrane domain is located between amino acids 500–522. V4 represents an apathogenic genotype I strain in the NDV vaccine strain, and LaSota corresponds to a lentogenic genotype II NDV vaccine strain. “–” denotes that no amino acid substitution was observed at the indicated residue.

For the HN protein, the cytoplasmic domain (amino acids 1–20), transmembrane domain (amino acids 21–49), stalk domain (amino acids 50–124), and neutralizing epitopes were examined (Table 3). In class I, sub-genotype 1.2 isolates, one substitution (A4G) was found in the cytoplasmic domain, three in the transmembrane domain (A28T, I29V, and E49D), and nine in the stalk domain (S54Q, V57E, T61V, R65K, E68D, S76A, N77S, V81I, and N120A). Neutralizing epitopes contained seven substitutions (N263Q, K333Q, E347D, D349E, I352V, I514V, and D569E).

**TABLE 3:** Amino acid substitutions in the HN protein of APMV-1 isolates.

Strain	Genotype	Cytoplasmic domain	Transmembrane domain			Stalk domain									Neutralizing epitopes					
		A^4^	A^28^	I^29^	E^49^	S^54^	V^57^	T^61^	R^65^	E^68^	S^76^	N^77^	V^81^	N^120^	^193^LS GCRD HSH^201^	N^263^	K^333^	^346^DEQ DYQ IR^353^	I^514^	D^569^
V4	Class I	–	–	–	–	–	–	–	–	–	–	–	–	–	–	–	–	R353Q	–	–
LaSota	Class II	–	–	–	–	–	–	–	–	–	–	–	–	S	–	–	–	–	–	–
20JBN-F12-31-35	Class I 1.2	G	T	V	D	G	E	V	K	D	A	S	I	A	–	Q	Q	E347D, D349E, I352V	V	E
20JBN-F12-41-45	Class I 1.2	G	T	V	D	Q	E	V	K	D	A	S	I	A	–	Q	Q	E347D, D349E, I352V, R353Q	V	E
20JBN-F12-46-50	Class I 1.2	G	T	V	D	Q	E	V	K	D	A	S	I	A	–	Q	Q	E347D, D349E, I352V	V	E
20JBN-F13-21-25	Class II I.2	–	–	–	–	Q	–	–	–	–	–	–	–	S	–	–	R	–	–	–
21JBN-F27-27	Class II I.2	–	–	–	–	–	–	–	–	–	–	–	–	S	–	–	–	–	–	–
21JBN-F1-50	Class II I.2	–	–	–	–	–	–	–	–	–	–	–	–	S	–	–	–	–	–	–
22JBN-F12-14	Class II I.2	–	–	–	–	–	–	–	–	–	–	–	–	S	S200P	–	R	–	–	–
22JBN-F2-10	Class I 1.2	G	T	V	D	–	E	V	K	D	A	S	I	A	–	Q	Q	E347D, D349E, I352V	V	E
23JBN-F22-66-70	Class I 1.2	G	T	V	N	Q	E	V	K	D	A	S	I	A	–	Q	Q	E347D, D349E, I352V	V	E
23JBN-F2-1	Class I 1.2	G	T	V	N	Q	E	V	K	D	A	S	I	A	–	Q	Q	E347D, D349E, I352V	V	E
23JBN-F7-11	Class II I.2	–	–	–	–	Q	–	–	–	–	–	–	–	–	–	–	–	–	–	–
23JBN-F7-68	Class I 1.2	G	A	V	N	N	E	V	K	D	A	S	I	A	-	Q	Q	E347D, D349E, I352V	V	G

The cytoplasmic domain is located in amino acids 1–20, the transmembrane domain 21–49, and the stalk domain 50–124. V4 represents an avirulent genotype I strain in the NDV vaccine strain, and LaSota corresponds to a lentogenic genotype II NDV vaccine strain. “–” denotes that no amino acid substitution was observed at the indicated residue. “–” indicates that no amino acid substitution was observed at the indicated residue.

In contrast, class II sub-genotype I.2 isolates showed no substitutions in the cytoplasmic or transmembrane domains. Only one substitution at position 120 (N120S) in the stalk domain was observed in class II sub-genotype I.2 isolates, except the 23JBN-F7-11. Additionally, a single substitution (K333R) was detected in the neutralizing epitope region of 20JBN-F13-21-25 and 22JBN-F12-14. Two unique amino acid substitutions were identified in individual strains, with K321R detected in 21JBN-F1-50 and S200P in 22JBN-F12-14.

### 3.4 Genetic and phylogenetic characterization of APMV-4

**FIGURE 3:**
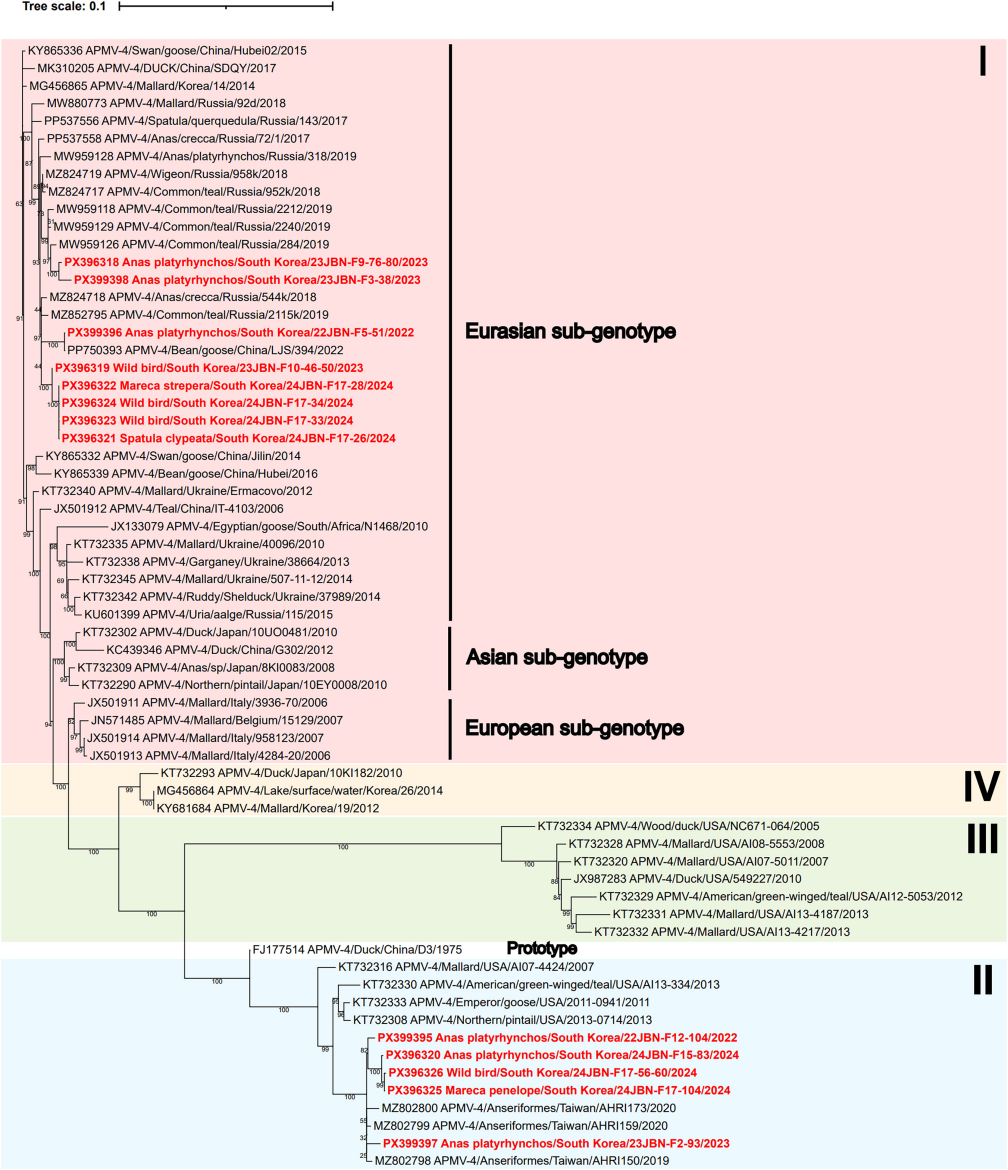
Phylogenetic tree based on the complete F nucleotide sequences of APMV-4. The phylogenetic tree was constructed using maximum likelihood with the K3Pu+F+I model and supported by 1,000 bootstrap replicates. APMV-4 strain sequences in the present study are highlighted in bold red.

A total of 13 APMV-4 isolates were obtained in this study. Phylogenetic analysis of the complete F gene revealed that two distinct genetic groups of APMV-4 are circulating among wild birds in South Korea. Eight APMV-4 isolates clustered within the Eurasian sub-genotype of Genotype I, while the remaining five APMV-4 isolates grouped into Genotype II (Figure 3). Among the Genotype I isolates, four (24JBN-F17-28, 24JBN-F17-34, 24JBN-F17-26, and 24JBN-F17-33) exhibited 100% nucleotide identity with each other. Together with 23JBN-F10-46-50, these four isolates showed the highest nucleotide similarity to the Wigeon/Novosibirsk region, Russia/958k/2018. 22JBN-F5-51 isolate shared complete identity with Bean goose/China/LJS 394/2022. Two isolates, 23JBN-F9-76-80 and 23JBN-F3-38, showed the highest similarity to Common teal/Primorje/Russia/284/2019 with 98.8% and 99.2% identity, respectively. The five genotype II isolates (22JBN-F12-104, 23JBN-F2-93, 24JBN-F15-83, 24JBN-F17-56-60, and 24JBN-F17-104) clustered together and were closely related to Anseriformes/Taiwan/AHRI159/2020, with nucleotide identities exceeding 99%.

The GS and GE signal sequences identified in APMV-4 isolates were 3’-UCCCACCCCUUCC-5’ and 3’-AAAUUAAU_5_-5’, respectively, consistent with those reported in previous studies (Nayak et al., 2008). All isolates shared the same deduced F protein cleavage site motif, DIQPRF, which is characteristic of avirulent strains. Three hypervariable regions of the F protein, comprising amino acid positions 147–189, 276–307, and 479–508, were analyzed, and only one amino acid substitution, S480N, was identified in isolates belonging to Genotype II (Zhang et al., 2021).

### 3.5 Genetic and phylogenetic characterization of APMV-6

**FIGURE 4:**
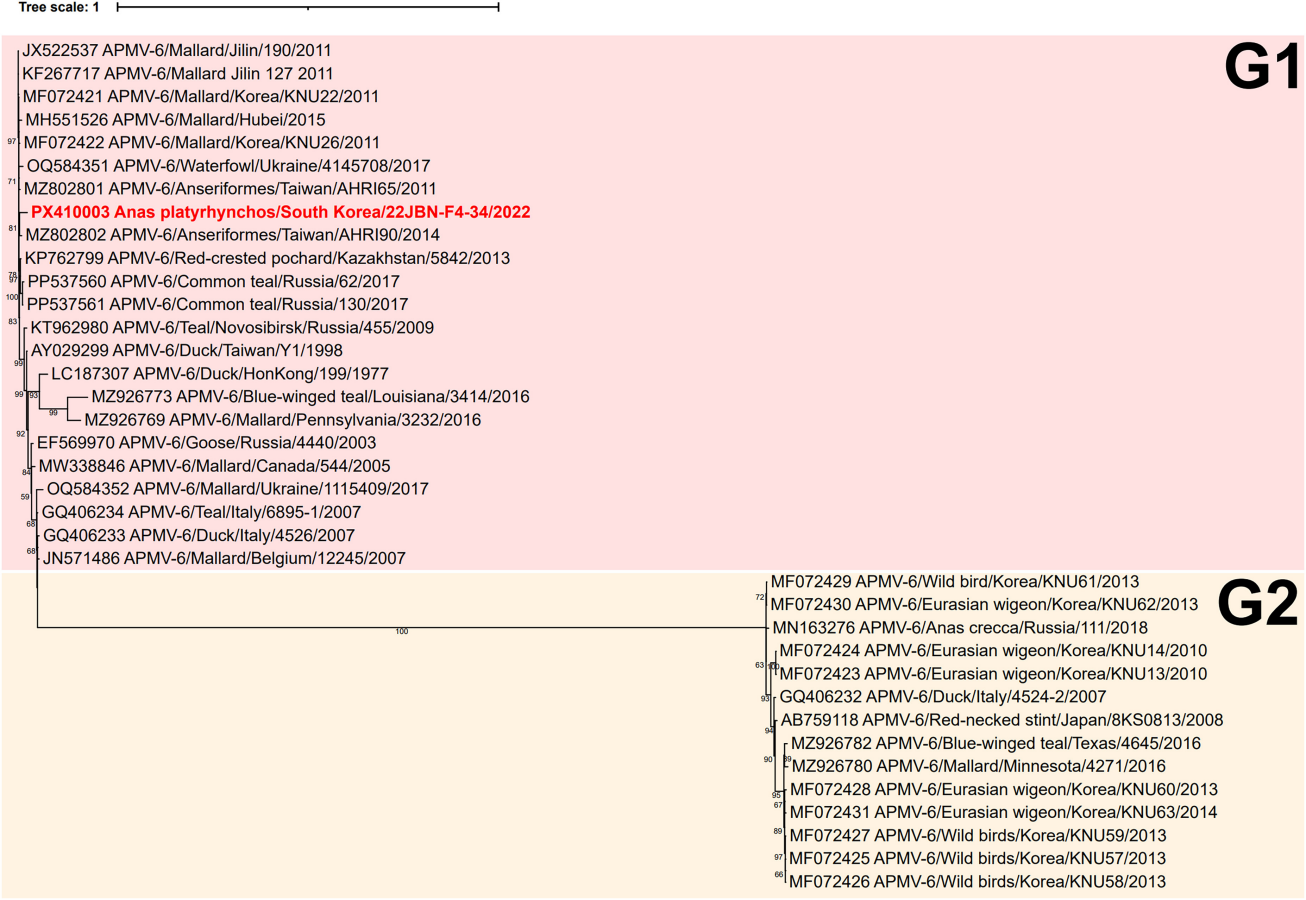
Phylogenetic tree based on the complete F nucleotide sequences of APMV-6. The phylogenetic tree was constructed using maximum likelihood with the HKY+F+I model and supported by 1,000 bootstrap replicates. The APMV-6 strain sequence in the present study is highlighted in bold red.

Phylogenetic analysis of the complete F gene revealed that one APMV-6 isolate, 22JBN-F4-34, collected from *Anas platyrhynchos* in 2022, was classified into genotype G1 (Figure 4). The 22JBN-F4-34 showed the highest nucleotide identity of 99.7% with the Mallard/South Korea/KNU22/2011. In addition, 22JBN-F4-34 exhibited high sequence similarity (≥ 96.8%) with APMV-6 isolates from wild birds across Eurasia, including China, Taiwan, Kazakhstan, and Russia (Supplementary Table 2).

Unlike other APMV serotypes, APMV-6 genomes contain an additional gene encoding the small hydrophobic (SH) protein, resulting in the gene order: 3’-N-P-M-F-SH-HN-L-5’ (Xiao et al., 2010). The GS and GE signal sequences identified in 22JBN-F4-34 were 3’-CUC_5_UUC-5’ and 3’-AAU(N_1–2_)AU_4–6_-5’, respectively (Samuel et al., 2010). The deduced cleavage site motif of the F protein was APEPRL, which is consistent with avirulent strains.

### 3.6 Genetic and phylogenetic characterization of APMV-13

**FIGURE 5:**
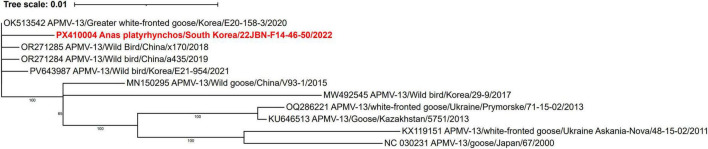
Phylogenetic tree based on the complete F nucleotide sequences of APMV-13. The phylogenetic tree was constructed using maximum likelihood with the TPM2+F model and supported by 1,000 bootstrap replicates. The APMV-13 strain sequence in the present study is highlighted in bold red.

Phylogenetic analysis based on the complete L genome sequence revealed that 22JBN-F14-46-50 was classified as APMV-13 (Figure 1). Complete F gene analysis showed that the 22JBN-F14-46-50 shared 99.6% nucleotide identity with the APMV-13 strain Greater white-fronted goose/South Korea/E20-158-3/2020 (Figure 5). The GS and GE signal sequences were identified as 3’-UCCCCGUCUU-5’ and 3’-U(C/A)U_6_-5’, respectively. The amino acid sequences at the F protein cleavage site were VRENRL, which is characteristic of avirulent strains. Genomic sequence analysis further revealed that 22JBN-F14-46-50 exhibited an 84-nucleotide deletion at positions 15304-15387 in the 5’ trailer region, which was also observed in reference strain E20-158-3 (Supplementary Figure 1).

### 3.7 Pathogenicity index test

The MDT for 21JBN-F27-27 was 89.6 h, corresponding to a mesogenic classification based on MDT criteria (Table 4). The remaining six APMV isolates were classified as lentogenic strains, with MDT beyond 168 h and possessing monobasic cleavage site motifs.

**TABLE 4:** Mean death time (MDT) of representative APMVs in embryonated eggs.

Strain	Serotype	Genotype	MDT (h)	Pathotypes
23JBN-F22-66-70	APMV-1	Class I 1.2 (1c)	> 168 h	Lentogenic
22JBN-F2-10	APMV-1	Class I 1.2 (1d)	> 168 h	Lentogenic
21JBN-F27-27	APMV-1	Class II I.2	89.6	Mesogenic
23JBN-F3-38	APMV-4	genotype I	> 168 h	Lentogenic
24JBN-F15-83	APMV-4	genotype II	> 168 h	Lentogenic
22JBN-F4-34	APMV-6	G1	> 168 h	Lentogenic
22JBN-F14-46-50	APMV-13	Not assigned	> 168 h	Lentogenic

Mean death time (MDT) indicates the mean time (hours) required for the minimum lethal dose (MLD) of virus to cause death in all inoculated embryos. Pathotypes were classified as velogenic (< 60 h), mesogenic (60–90 h), and lentogenic strains (> 90 h).

### 3.8 Antigenic cross-reactivity with NDV

All 12 APMV-1 isolates identified in this study were evaluated for antigenic cross-reactivity with the NDV vaccine strain using a HI assay with reference anti-NDV LaSota serum. All isolates exhibited low HI titers (≤ 20).

## 4 Discussion

This study expands our understanding of the genetic diversity and molecular characteristics of APMVs circulating in migratory wild birds in South Korea between 2020 and 2024. A total of 27 APMV isolates were obtained from wild birds during the winter seasons between 2020 and 2024, with a mean isolation rate of 0.35%. This result is comparable to a previous study from South Korea, which reported a mean APMV isolation rate of 0.4% during the winter seasons from 2015 to 2021 (Lee et al., 2024). All isolates were derived from *Anseriformes* species, which aligns with previous studies and demonstrates that these birds serve as a natural reservoir of APMVs (Gulyaeva et al., 2022; Cross et al., 2013). These findings highlight the importance of continued surveillance efforts targeting *Anseriformes* species to improve understanding of the ecology and evolution of APMVs.

Four distinct APMV serotypes were identified, and coding-complete genome sequences were obtained using a SISPA approach. Recent advances in high-throughput sequencing technologies have markedly improved the availability of complete genome data for previously underrepresented APMV serotypes (Van Borm et al., 2023). SISPA-based next-generation sequencing has been effectively applied in previous studies to generate complete APMV genomes and was similarly employed in this investigation (Chrzastek et al., 2017; Kariithi et al., 2022; Cho et al., 2022). However, the 5’ and 3’ UTRs were only partially recovered in most isolates. This limitation is consistent with a previous study reporting that SISPA-based approaches often fail to resolve the terminal 100-200 nucleotides of viral UTRs (Tal et al., 2019). In this study, the limited sequencing depth (approximately 100,000 reads per sample) may have contributed to the insufficient coverage required for complete UTR recovery. To enhance the recovery of full-length UTRs, the use of NGS platforms with higher read output or the incorporation of rapid amplification of cDNA ends (RACE)-PCR might be beneficial.

APMV-1 continues to receive particular attention due to its established pathogenic potential and significant implications for the poultry industry. Newcastle disease, caused by virulent strains of APMV-1, was first reported in South Korea in 1927 and has remained a significant concern for the national poultry industry (Kim and Song, 1992). Although no outbreaks have been recorded in domestic poultry farms in South Korea in recent years, APMV-1 is still regularly detected in wild bird populations (Choi et al., 2012; Lee et al., 2024). In the present study, 12 APMV-1 isolates were obtained and classified into class I sub-genotype 1.2 and class II sub-genotype I.2. Based on the classification system proposed by Diel et al. (2012), the class I sub-genotype 1.2 viruses were further divided into sub-genotype 1c and 1d (Diel et al., 2012). While sub-genotype 1d has predominantly been reported in North America, sub-genotype 1c includes strains originating from diverse regions, including Europe, East Asia, and Central Asia (Hicks et al., 2019). However, our findings suggest that sub-genotype 1d is not limited to North America. In particular, 23JBN-F22-66-70, classified as sub-genotype 1d, showed high nucleotide similarity to East Asian strains, indicating that this lineage also includes viruses of East Asian origin. This observation suggests the ongoing intercontinental circulation driven by migratory bird movements. Although Class II sub-genotype I.2 strains are typically lentogenic and detected in wild waterfowl, possible transmission to domestic poultry has been suggested in previous studies (Lee et al., 2024; Reid et al., 2023). In this study, no evidence of Class II sub-genotype I.2 virus transmission from wild waterfowl to domestic poultry was identified, although careful monitoring remains essential.

All 12 APMV-1 isolates identified in this study possessed a monobasic cleavage site in the F protein. Representative class I sub-genotype 1.2 isolates, including 23JBN-F22-66-70 and 22JBN-F2-10, exhibited MDT of > 168 h, which is consistent with the presence of a monobasic cleavage site. In contrast, 21JBN-F27-27 exhibited MDT of 89.6 h and was classified as mesogenic despite also possessing a monobasic cleavage site. This finding contrasts with the general understanding that mesogenic NDVs have a polybasic cleavage site motif within the F protein (Xiao et al., 2012). These findings suggest that MDT-based pathogenicity does not always strictly correlate with the F protein cleavage site motif. Although MDT alone is insufficient for assessing viral pathogenicity and additional animal experiments would be required, the higher MDT-based pathogenicity observed for 21JBN-F27-27 relative to other APMV-1 isolates suggests that class II sub-genotype 1.2 viruses warrant further investigation in surveillance and pathogenicity studies.

Lentogenic live vaccines based primarily on genotypes I and II are currently widely employed worldwide for Newcastle disease control. The F and HN amino acid sequences of the isolated APMV-1 strains were compared with those of representative vaccine strains, including V4 (genotype I) and LaSota (genotype II), both of which are commonly used as lentogenic live vaccines (Hu et al., 2022). Considering the crucial role of F and HN proteins in eliciting protective immune responses, identifying amino acid differences in key functional domains may provide insight into potential antigenic variation and implications for vaccine efficacy (Kumar et al., 2011). The results showed that while class II sub-genotype I.2 isolates exhibited conserved amino acid sequences, class I sub-genotype 1.2 isolates exhibited notable amino acid diversity, especially within the F protein. Amino acid substitutions in the F gene have been suggested to influence antigenic properties, which may have implications for current vaccine performance (Shahar et al., 2018). In support of this, antigenic cross-reactivity assays using anti-NDV LaSota serum demonstrated uniformly low HI titers (≤ 20) against class I sub-genotype 1.2 isolates. Notably, low HI titers were also observed for class II sub-genotype I.2 isolates despite their relatively conserved F and HN amino acid sequences, indicating limited antigenic cross-reactivity with the NDV vaccine strain. Although these findings suggest antigenic divergence between wild bird-derived APMV-1 isolates and commonly used NDV vaccine strain, further studies, including virus neutralization assays and *in vivo* vaccination—challenge experiments, are required to determine whether currently commercialized vaccines provide effective protection against APMV-1 isolates originating from wild birds.

According to a recent phylogenetic classification based on MCC tree analyses of the F gene, APMV-4 is grouped into four major genotypes (I–IV), with Genotype I further subclassified into three distinct sub-genotypes (Zhang et al., 2021). Previously identified APMV-4 isolates in South Korea were predominantly classified into Genotype I (formerly referred to as the Old-World I genotype), which includes both Eurasian and Asian sub-genotypes, as well as Genotype IV (formerly referred to as the Old-World II genotype). In this study, APMV-4 strains belonging to both the Eurasian sub-genotype of Genotype I and to Genotype II were identified. The APMV-4 isolates exhibited high nucleotide similarity to strains previously reported in China, Russia, and Taiwan. Notably, five APMV-4 isolates belonged to Genotype II, a lineage referred to as the New-World I genotype, which includes APMVs primarily isolated from wild birds in the Americas. To date, no Genotype II APMV-4 strains have been reported from wild birds in South Korea. The identification of Genotype II APMV-4 strains in this study suggests potential interhemispheric exchange between the Eastern and Western Hemispheres, with novel APMV-4 genotypes that might be introduced into South Korea through migratory flyways.

The cleavage sites of all APMV-4 F genes lacked multiple basic residues, indicating lentogenic viruses. In addition, consistent with a previous study, the hypervariable regions of the F protein in APMV-4 isolates from this study were relatively conserved (Zhang et al., 2021). However, APMV-4 has been documented not only in wild birds but also in domestic poultry such as ducks, geese, and chickens (Zhang et al., 2021; Yin et al., 2017; Tseren-Ochir et al., 2017). In addition, given the potential for APMV-4 transmission from wild birds to domestic poultry, continued surveillance is warranted in South Korea, even when APMV-4 is detected only in wild bird populations (Yin et al., 2017).

APMV-6 has been classified into two genotypes, G1 and G2, based on phylogenetic analysis (Choi et al., 2018; Young et al., 2022). Interestingly, G2 shows substantial genetic divergence from G1 and has even been proposed as a putative novel species cluster (Young et al., 2022). In South Korea, the two genotypes have also demonstrated distinctive ecological characteristics (Choi et al., 2018). In the present study, one APMV-6 strain (22JBN-F4-34) belonging to the G1 genotype was isolated and characterized by a SH protein that distinguishes it from other APMV serotypes. 22JBN-F4-34 showed high nucleotide similarity to isolates from wild birds in neighboring countries such as China, Taiwan, and Russia, suggesting possible cross-regional circulation of APMV-6.

Nevertheless, due to the limited availability of genetic data, a comprehensive phylogenetic relationship between the genotypes G1 and G2 remains unclear. Sequence analysis of the F gene cleavage site in 22JBN-F4-34 indicated a lentogenic APMV. Nonetheless, experimental evidence from an APMV-6 strain isolated in South Korea has demonstrated its ability to infect poultry and shed virus, highlighting the importance of sustained monitoring efforts (Lee et al., 2013).

APMV-13 is known to circulate at low levels among wild bird populations and has been reported less frequently compared to other APMV serotypes (Lee et al., 2024). The 22JBN-F14-46-50 exhibited the highest nucleotide similarity to a previously reported strain, Greater white-fronted goose/South Korea/E20-158-3/2020. Consistent with other APMV-13 strains, 22JBN-F14-46-50 possessed a lentogenic-type fusion protein cleavage site motif (VRENRL). Notably, the 22JBN-F14-46-50 also exhibited an 84-nucleotide deletion at positions 15,304–15,387, which was identical to that observed in the E20-158-3 strain (Cho et al., 2022). Furthermore, an identical deletion was also identified in another Korean isolate, E21-954 (accession number: PV643987), detected in a wild bird in Korea in 2021. These results suggest the possibility of emerging genomic diversity within APMV-13 strains currently circulating in South Korea. However, due to the limited availability of full-length genomic sequences of APMV-13, further studies are needed to clarify the evolution and genetic diversity of APMV-13.

APMVs have been continuously reported in migratory wild birds in South Korea, including a variety of subtypes and even novel serotypes (Lee et al., 2024; Lee et al., 2017). In the present study, we provide the first molecular evidence of a genotype II APMV-4 strain in wild birds from South Korea, thereby expanding the current understanding of the genetic diversity and evolutionary complexity of APMVs circulating in East Asia. In addition, class I sub-genotype 1.2 of APMV-1 isolates identified in this study exhibited multiple amino acid substitutions in the F protein when compared with commonly used NDV vaccine strains, which may have implications for vaccine efficacy. These results highlight the importance of ongoing surveillance of APMV-1 strains circulating in wild bird populations. Moreover, regardless of serotype, phylogenetic studies provide evidence of active intercontinental exchanges of APMVs. The nearly complete nucleotide sequences obtained from diverse APMV subtypes provide valuable information that will contribute to future investigations into the genetic evolution, antigenic variability, and epidemiological dynamics of APMVs.

## Data Availability

The datasets presented in this study can be found in online repositories. The names of the repository/repositories and accession number(s) can be found in this article/Supplementary material.
