# Supplementary material for: Genetic diversity and molecular characterization of avian paramyxoviruses from wild birds in South Korea between 2020 and 2024
Source: Front Microbiol. 2026 Feb 17;17:1732075. doi: 10.3389/fmicb.2026.1732075 (PMC12953492; doi:10.3389/fmicb.2026.1732075)
Supplement: Supplementary file 1 [file Table_1.docx]

Supplementary Material

***
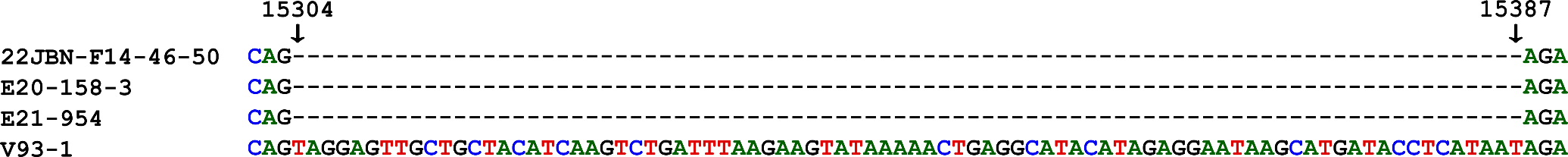
***

# Supplementary Figure 1

Nucleotide sequence alignment of the 5′ trailer region of APMV-13 isolates highlighting an 84-nucleotide deletion in 22JBN-F14-46-50 compared with the reference APMV-13 strains. The reference sequences included E20-158-3 (OK513542; APMV-13/Greater white-fronted goose/South Korea/E20-158-3/2020), E21-954 (PV643987; APMV-13/Wild bird/South Korea/E21-954/2021), and V93-1 (MN150295; APMV-13/Wild goose/China/V93-1/2015).

# Supplementary Table 1

Genome features and sequence lengths of APMVs isolated in this study.

Numbers in the parentheses indicate the lengths of non-translated upstream and downstream regions of each gene, and bold values represent the lengths of coding regions. An asterisk (*) denotes partial sequences, and a blank indicates sequences that could not be determined. NP, Nucleotide protein; P, phosphoprotein; M, matrix protein; F, fusion protein; HN, hemagglutinin-neuraminidase; L, large polymerase; SH, small hydrophobic protein. The SH protein is exclusively present in APMV-6, which follows the gene order 3'-N-P-M-F-SH-HN-L-5'.

IGS, non-coding intergenic sequence.

| **Isolates** | **Serotype** | **3' leader** | **NP** | **NP/P IGS** | **P** | **P/M IGS** | **M** | **M/F IGS** | **F** | **F/HN IGS** | **HN** | **HN/L**  **IGS** | **L** | **5' trailer** |
| --- | --- | --- | --- | --- | --- | --- | --- | --- | --- | --- | --- | --- | --- | --- |
| **20JBN-F12-31-35** | APMV-1 | 48* | (66)**1470**(211) | 2 | (83)**1200**(180) | 1 | (34)**1095**(112) | 1 | (46)**1662**(84) | 31 | (91)**1851**(59) | 48 | (11)**6615**(77) | 109 |
| **20JBN-F12-41-45** | APMV-1 |  | **78***(209) | 2 | (83)**1200**(180) | 1 | (34)**1095**(112) | 1 | (46)**1662**(84) | 31 | (91)**1851**(59) | 48 | (11)**6615**(77) | 59* |
| **20JBN-F12-46-50** | APMV-1 | 55 | (55*)**1470**(209) | 2 | (83)**1200**(180) | 1 | (34)**1095**(112) | 1 | (46)**1662**(84) | 31 | (91)**1851**(59) | 48 | (11)**6615**(77) | 66* |
| **20JBN-F13-21-25** | APMV-1 | 48 | (66)**1470**(210) | 2 | (83)**1188**(180) | 1 | (34)**1095**(112) | 1 | (46)**1662**(84) | 31 | (91)**1851**(60) | 47 | (11)**6615**(77) | 113* |
| **21JBN-F27-27** | APMV-1 | 49* | (66)**1470**(210) | 2 | (83)**1188**(180) | 1 | (34)**1095**(112) | 1 | (46)**1662**(84) | 31 | (91)**1851**(60) | 47 | (11)**6615**(77) | 107 |
| **21JBN-F1-50** | APMV-1 |  | (63)**1470**(210) | 2 | (83)**1188**(180) | 1 | (34)**1095**(112) | 1 | (46)**1662**(84) | 31 | (91)**1851**(60) | 47 | (11)**6615**(77) | 115 |
| **22JBN-F12-14** | APMV-1 |  | (58*)**1470**(210) | 2 | (83)**1188**(180) | 1 | (34)**1095**(112) | 1 | (46)**1662**(84) | 31 | (91)**1851**(60) | 47 | (11)**6615**(77) | 114 |
| **22JBN-F12-104** | APMV-4 | 51 | (60)**1374**(117) | 9 | (46)**1182**(136) | 34 | (77)**1110**(106) | 13 | (74)**1701**(116) | 37 | (69)**1710**(135) | 42 | (95)**6636**(64*) | - |
| **22JB-F2-10** | APMV-1 | 31* | (66)**1470**(210) | 2 | (83)**1200**(180) | 1 | (34)**1095**(112) | 1 | (46)**1662**(84) | 31 | (91)**1851**(60) | 47 | (11)**6615**(77) | 107* |
| **22JBN-F4-34** | APMV-6 | 49* | (72)**1398**(101) | 7 | (53)**1293**(140) | 2 | (113)**1101**(191) | 59 | (12)**1668**(156) | (F/SH IGS)  48  (SH/HN IGS)  28 | SH (72)**429**(73)  HN (50)**1842**(139) | 63 | (112)**6726**(179) | 19 |
| **22JBN-F5-51** | APMV-4 | 36* | (60)**1374**(117) | 9 | (46)**1182**(136) | 34 | (77)**1110**(106) | 13 | (74)**1701**(116) | 37 | (69)**1710**(135) | 42 | (95)**6636**(64*) |  |
| **22JBN-F14-46-50** | APMV-13 | 54 | (60)**1482**(179) | 14 | (95)**1194**(223) | 1 | (34)**1101**(200) | 2 | (45)**1638**(177) | 12 | (92)**1833**(145) | 25 | (13)**6600**(469*) |  |
| **23JBN-F22-66-70** | APMV-1 | 49* | (66)**1470**(210) | 2 | (83)**1200**(180) | 1 | (34)**1095**(112) | 1 | (46)**1662**(84) | 31 | (91)**1851**(60) | 48 | (11)**6615**(77) | 102* |
| **23JBN-F2-1** | APMV-1 |  | (18*)**1470**(210) | 2 | (83)**1200**(180) | 1 | (34)**1095**(112) | 1 | (46)**1662**(84) | 31 | (91)**1851**(59) | 48 | (11)**6615**(77) | 114 |
| **23JBN-F2-93** | APMV-4 | 46 | (60)**1374**(117) | 9 | (46)**1182**(136) | 34 | (77)**1110**(106) | 13 | (74)**1701**(116) | 37 | (69)**1710**(135) | 42 | (95)**6636**(75*) |  |
| **23JBN-F3-38** | APMV-4 | 55 | (60)**1374**(117) | 9 | (46)**1182**(136) | 34 | (77)**1113**(103) | 13 | (74)**1701**(116) | 37 | (81)**1698**(135) | 42 | (95)**6636**(103) | 13* |
| **23JBN-F7-11** | APMV-1 |  | (13*)**1470**(210) | 2 | (83)**1188**(180) | 1 | (34)**1095**(112) | 1 | (46)**1662**(84) | 31 | (91)**1851**(60) | 47 | (11)**6615**(77) | 83* |
| **23JBN-F7-68** | APMV-1 | 39* | (66)**1470**(210) | 2 | (83)**1200**(180) | 1 | (34)**1095**(112) | 1 | (46)**1662**(84) | 31 | (91)**1851**(59) | 48 | (11)**6615**(77) | 65 |
| **23JBN-F9-76-80** | APMV-4 | 12* | (60)**1374**(117) | 9 | (46)**1182**(136) | 34 | (77)**1113**(103) | 13 | (74)**1701**(116) | 37 | (81)**1698**(135) | 42 | (95)**6608*** |  |
| **23JBN-F10-46-50** | APMV-4 | 49* | (60)**1374**(117) | 9 | (46)**1182**(136) | 34 | (77)**1113**(103) | 13 | (74)**1701**(116) | 37 | (81)**1698**(135) | 42 | (95)**6636**(103) | 17 |
| **24JBN-F15-83** | APMV-4 | 55 | (60)**1374**(117) | 9 | (46)**1182**(136) | 34 | (77)**1110**(106) | 13 | (74)**1701**(116) | 37 | (69)**1710**(135) | 42 | (95)**6636**(103) | 17 |
| **24JBN-F17-26** | APMV-4 | 55 | (60)**1374**(117) | 9 | (46)**1182**(136) | 34 | (77)**1113**(103) | 13 | (74)**1701**(116) | 37 | (81)**1698**(135) | 42 | (77)**6636**(103) | 17 |
| **24JBN-F17-28** | APMV-4 | 49* | (60)**1374**(117) | 9 | (46)**1182**(136) | 34 | (77)**1113**(103) | 13 | (74)**1701**(116) | 37 | (81)**1698**(135) | 42 | (77)**6636**(103) | 21 |
| **24JBN-F17-33** | APMV-4 | 47* | (60)**1374**(117) | 9 | (46)**1182**(136) | 34 | (77)**1113**(103) | 13 | (74)**1701**(116) | 37 | (81)**1698**(135) | 42 | (95)**6636**(103) | 28 |
| **24JBN-F17-34** | APMV-4 | 49* | (60)**1374**(117) | 9 | (46)**1182**(136) | 34 | (77)**1113**(103) | 13 | (74)**1701**(116) | 37 | (81)**1698**(135) | 42 | (95)**6636**(103) | 10 |
| **24JBN-F17-104** | APMV-4 | 39* | (60)**1374**(117) | 9 | (46)**1182**(136) | 34 | (77)**1110**(106) | 13 | (74)**1701**(116) | 37 | (81)**1698**(135) | 42 | (95)**6636**(103) | 17 |
| **24JBN-F17-56-60** | APMV-4 | 55 | (60)**1374**(117) | 9 | (46)**1182**(136) | 34 | (77)**1110**(106) | 13 | (74)**1701**(116) | 37 | (81)**1698**(135) | 42 | (95)**6636**(103) | 10 |

# Supplementary Table 2

Pairwise nucleotide identity (%)based on complete F gene sequences between 22JBN-F4-34 and reference APMV-6 strains.

| **Isolates** | **Accession number** | **Nucleotide identity (%)** |
| --- | --- | --- |
| APMV-6/Mallard/Jilin/127/2011 | KF267717 | 97.6 |
| APMV-6/Mallard/Jilin/190/2011 | JX522537 | 97.6 |
| APMV-6/Duck/Korea/KNU22/2011 | MF072421 | 97.7 |
| APMV-6/Duck/Korea/KNU26/2011 | MF072422 | 97.6 |
| APMV-6/Mallard/Hubei/2015 | MH551526 | 97.3 |
| APMV-6/Red-crested pochard/Kazakhstan/5842/2013 | KP762799 | 97.3 |
| APMV-6/Anseriformes/Taiwan/AHRI90/2014 | MZ802802 | 97.5 |
| APMV-6/Anseriformes/Taiwan/AHRI65/2011 | MZ802801 | 97.6 |
| APMV-6/common teal/Dagestan/Russia/130/2017 | PP537561 | 97.1 |
| APMV-6/common teal/Dagestan/Russia/62/2017 | PP537560 | 96.8 |
